# Context Fear Conditioning in Down Syndrome Mouse Models: Effects of Trisomic Gene Content, Age, Sex and Genetic Background

**DOI:** 10.3390/genes12101528

**Published:** 2021-09-28

**Authors:** Md. Mahiuddin Ahmed, Aaron Block, Nicolas Busquet, Katheleen J. Gardiner

**Affiliations:** 1 Department of Neurology, Linda Crnic Institute for Down Syndrome, University of Colorado Alzheimer’s and Cognition Center, Aurora, CO 80045, USA; md.mahiuddin.ahmed@cuanschutz.edu; 2 Department of Pediatrics, University of Colorado Anschutz Medical Campus, Aurora, CO 80045, USA; aaroncblock@gmail.com; 3 Department of Neurology, Animal Behavior and In Vivo Neurophysiology Core, NeuroTechnology Center, University of Colorado Anschutz Medical Campus, Aurora, CO 80045, USA; nicolas.busquet@cuanschutz.edu

**Keywords:** down syndrome mouse model, hippocampus, mouse chromosome 16, 17, 10, Dp(10)1Yey, associative learning, sex differences

## Abstract

Down syndrome (DS), trisomy of the long arm of human chromosome 21 (Hsa21), is the most common genetic cause of intellectual disability (ID). Currently, there are no effective pharmacotherapies. The success of clinical trials to improve cognition depends in part on the design of preclinical evaluations in mouse models. To broaden understanding of the common limitations of experiments in learning and memory, we report performance in context fear conditioning (CFC) in three mouse models of DS, the Dp(16)1Yey, Dp(17)1Yey and Dp(10)1Yey (abbreviated Dp16, Dp17 and Dp10), separately trisomic for the human Hsa21 orthologs mapping to mouse chromosomes 16, 17 and 10, respectively. We examined female and male mice of the three lines on the standard C57BL/6J background at 3 months of age and Dp17 and Dp10 at 18 months of age. We also examined female and male mice of Dp17 and Dp10 at 3 months of age as F1 hybrids obtained from a cross with the DBA/2J background. Results indicate that genotype, sex, age and genetic background affect CFC performance. These data support the need to use both female and male mice, trisomy of sets of all Hsa21 orthologs, and additional ages and genetic backgrounds to improve the reliability of preclinical evaluations of drugs for ID in DS.

## 1. Introduction

Down syndrome (DS) is caused by an extra copy of all or, rarely, part of the long arm of human chromosome 21 (Hsa21). All individuals with DS display some level of intellectual disability (ID) [1,2], and although the severity of cognitive impairment varies among individuals, and can be mild, the average IQ is in the range of 40–50. DS is thus a significant medical and social condition given that the worldwide incidence is approximately one in 700–1000 live births [3,4,5] and because life expectancy in many countries is now ~60 years [4,6,7,8,9,10,11]. Lessening the severity of ID, even by 10–20 IQ points, would lead to increased independence for people with DS and expanded opportunities for education, employment and societal inclusion. Over the last several years, interest has grown in conducting clinical trials with pharmacotherapies to lessen or prevent ID in DS [12,13]. Several trials have been completed and more are proposed. None, however, produced significant, practical, positive outcomes for participants (ClinicalTrials.gov: NCT01576705, NCT02304302, NCT02024789, NCT02484703; [14,15]). Many reasons for these failures can be considered, including number and age of the participants and duration of the trial. However, another consideration is that, prior to clinical trials, candidate drugs most commonly are first evaluated in mice. As with other human diseases, the experimental design of such preclincal evaluations influences their reliability in prediction of outcomes for patients (reviewed in [16]).

There are two challenges to modeling DS in mice. First, DS is a contiguous gene syndrome, spanning the entirety of Hsa21q. While a DS Critical Region (DSCR) [17,18] was popularized as necessary and sufficient, suggesting that genes outside this region could be ignored, individuals have been diagnosed with DS who are trisomic for segments of Hsa21q that do not overlap with the DSCR [19,20]. Thus, the entirety of Hsa21q properly remains in consideration for contributions to the DS phenotype. Hsa21q encodes approximately 160 protein coding genes of diverse functions, an additional ~45 members of the family of Keratin Associated Proteins (KRTAPs), and many transcripts that may or may not be functional RNAs [21,22]. A small number of Hsa21 genes has been studied extensively for biological function and potential roles in trisomy, but for the large majority, little or nothing is known about their function [22]. Lacking such knowledge, no Hsa21 gene can be dismissed as irrelevant to the cognitive phenotype and therefore, the ideal genetic representation of human DS in mice remains trisomy of all genes.

The second challenge for modeling DS in mice is that orthologs of Hsa21 genes are present on segments of three mouse chromosomes, Mmu16, 17 and 10 [23]. There are technical challenges to generating complete trisomy, and while many partial trisomies have been created (reviewed in [21,24,25,26,27]), a full trisomy model has only recently been produced [28]. The oldest and most popular DS model is the Ts65Dn. It is trisomic for most of the Mmu16 orthologous region, encompassing 90 of the 160 Hsa21 non-KRTAP orthologs [21,23,29,30] and for ~30 protein coding genes (excluding pseudogenes) that map to a non-Hsa21 orthologous segment of Mmu17, irrelevant to DS [31]. In spite of its genetic shortcomings, the Ts65Dn has been shown to display a number of DS relevant neurological phenotypic features, including reduced sizes of the hippocampus and cerebellum, abnormal neuronal densities and morphologies, and impaired learning and memory in tasks requiring a functional hippocampus (reviewed in [25,26]). To date, clinical trials for cognition in DS have proceeded based on preclinical data obtained solely from the Ts65Dn [14,15,16,32]. Because of breeding characteristics, preclinical evaluations have used almost exclusively male mice [33]. These evaluations thus leave unexplored both the contributions to phenotype and drug responses of the ~40% of Hsa21 orthologs not trisomic in the Ts65Dn, as well as possible differences in responses in female mice. Lastly, again because of challenges in breeding large numbers of mice, most often a single age is tested, and although the Ts65Dn is on a mixed background, no effects of different genetic backgrounds have been explored.

Chromosomal engineering has more recently been used to create segmental duplications of the individual chromosomal regions of Mmu 16, 17 and 10 (reviewed in [26]). Among the resultant lines are the Dp(16)1Yey, the Dp(17)1Yey and the Dp(10)1Yey (abbreviated here as the Dp16, Dp17 and Dp10, respectively), which are trisomic for the entireties of the respective Hsa21 orthologous mouse regions [34]. Together, these can be used to investigate trisomy of all Hsa21 orthologous genes [35]. Young male mice (2–4 months of age) of the Dp16 line have been shown to be impaired in novel object recognition (NOR) and context fear conditioning (CFC) and to have repressed long term potentiation (LTP); the Dp17, to have normal CFC and NOR learning and elevated LTP; and the Dp10, to have normal learning in these tasks and normal LTP [34]. Older (3–7 months) Dp16 have been shown to be impaired in both CFC and the Morris Water Maze (MWM) [36]. The Dp10 have been shown to be impaired in a spontaneous alternation task at 3, 6 and 9 months [37]. Although these lines breed well, to date there have been no studies of learning/memory in female mice, and no studies with older mice or of mice on a genetic background other than the original C57BL/6J (or related C57BL/6 strains). Experiments to create the complete trisomy, i.e., the triple duplication, created by breeding the Dp17 with the Dp10, and then crossing the offspring that are double trisomics with the Dp16, have been reported [35,38]. These experiments are expensive in terms of time and resources. Here, we instead examined the three lines separately, to evaluate performance in CFC of separate cohorts of female and male mice at 3 months of age, of the Dp17 and Dp10 lines at 18 months of age and at 3 months of age on an F1 hybrid background obtained by crossing trisomic mice with controls from the DBA/2J inbred background. We found that aspects of CFC performance are influenced by specific trisomy, sex, age and genetic background.

## 2. Materials and Methods

### 2.1. Experimental Animals

Colonies of Dp(16)1Yey, Dp(17)1Yey and Dp(10)1Yey (abbreviated Dp16, Dp17 and Dp10, respectively) were established from breeding pairs obtained from YE Yu and maintained by crossing with C57BL/6J wild type mice obtained from The Jackson Laboratory. A colony of DBA/2J was established from breeding pairs obtained from The Jackson Laboratory. All mice for these experiments were bred at the University of Colorado School of Medicine (Aurora, CO, USA). Colonies were maintained in a room with HEPA-filtered air and a 14:10 light:dark cycle and, fed a 6% fat diet and acidified (pH 2.5–3.0) water ad libitum. Littermates (Supplementary Tables S1 and S2) of the same sex were housed in the same cage. Dp16, Dp17 and Dp10 mice were genotyped by quantitative (real time) polymerase chain reaction (qPCR) for genes in the trisomic segments as recommended by The Jackson Laboratory [39]. Separate cohorts of female and male mice were used in all experiments. Female mice were in diestrus.

### 2.2. Context Fear Conditioning

Context fear conditioning (CFC) was performed as described in [40,41]. Briefly, in a context-shock (CS) training session, mice were placed in a novel cage (Med Associates, St. Albans, VT, USA, Modular Mouse Test Chamber), allowed to explore for three minutes and then given a single electric shock (2 s, 0.7 mA, constant electric current). In a shock-context (SC) training session, a second group of mice were placed in the novel cage, immediately given the electric shock (2 s), and then allowed to explore for 3 min. Mice of both theCS and SC groups were returned to their home cages and 60 min later were, re-exposed to the same context for 3 min without any electric shock. In these testing sessions (CS-t and SC-t respectively), the time spent “freezing” (the absence of movement except for respiration) was measured. Freezing is indicative of associative learning between the aversive experience (shock) and the context in which the shock was received; in normal control mice, freezing is significantly higher in the CS group.

Prior to CFC, mice were handled for 2–3 min per day for 3 days. Immediately prior to assessment, mice were allowed to habituate to the behavior room for 30 min in their home cage. All experiments used separate cohorts of female and male mice of each genoytpe. Three sets of experiments were conducted. Experiment 1 used 3 month old mice, controls and trisomics, from the Dp16, Dp17 and Dp10 lines. Experiment 2 used 3 month old control mice from the DBA/2J inbred line, and F1 hybrid offspring from crossing female DBA/2J mice with (i) trisomic male Dp17 mice and (ii) trisomic male Dp10 mice. Experiment 3 used ~18-month-old mice (age range, 16–24 months), controls and trisomics, from the Dp17 and Dp10 lines. For this experiment, a no-shock (NS) group was added in which mice were similarly exposed to the novel context in a training and a test session, but never received an electric shock. Because of difficulties with breeding, Dp16 mice were not available for Experiments 2 and 3. In each experiment, an average of 10 mice per group was used; power analysis indicates that this is adequate, based on results reported in the literature for CFC and our experience with the DS lines, for detection of a 20% difference, with *p* < 0.05 and 15% standard deviation, with a power of 0.909. Entire litters were used at one time. In order to balance littermates between SC and CS groups, it was not possible for the experimentor to be blind to the sex/genotype of the mice. However, data are generated automatically by the FreezeScan software, version RRID:SCR_014495 (CleverSys, Reston, VA, USA) which precludes experimentor bias. Table 1 lists the number of mice in the SC and CS (and NS) groups, by genotype and sex, for each experiment. A total of 671 mice were used; four outliers were removed from analysis. Information for all mice regarding age and littermates is included in Supplemental Tables S1–S3.

### 2.3. Statistics

Statistical analyses were carried out using Prism 8.0 software (GraphPad, San Diego, CA, USA). Results were reported for each mouse as the number of seconds spent freezing in each 3 min session and during each 30 s interval of the 3 min session. The mean of seconds spent freezing of all mice within a single treatment, sex and genotype group was used in statistical calculations. Significant differences between groups were determined by unpaired Student’s t-test with SEM reported; *p* ≤ 0.05 was considered for statistical significance.

## 3. Results

### 3.1. Performance in CFC at 3 Months of Age (Experiment 1)

For each of the three partial trisomies, the Dp16, Dp17 and Dp10, cohorts of female and male mice, controls and trisomics, were analyzed separately. Time spent freezing in a novel context was measured before and after exposure to the electric shock. Measurements of freezing were made in two sessions: a training session where the mouse received a shock, was allowed to explore, and then was returned to the home cage for 60 min, followed by a testing session where the mouse was returned to the novel context but received no shock. Mice of each sex and genotype were separated into two groups: shock-context (SC), in which mice received the shock in the training session immediately upon being placed in the novel cage, and context-shock (CS), in which mice received the shock in the training session three minutes after being placed in the novel cage. Table 2 shows the average time freezing for each genotype/sex and CFC group.

Levels of freezing in SC and SC-t are similar within each line for each sex and genotype, and there are no significant differences between lines (including Dp17, in spite of levels >30 s). The lowest levels of freezing, ~7”–15”, occur in the CS training sessions (with the one exception of trisomic male Dp17). These reflect normal levels of locomotion in a novel environment because the electric shock is delivered at the end of the 3 min. The highest levels of freezing occur in the CS-t sessions, manifesting in normal learning in control mice where the novel cage is associated with the aversive electric shock. Control mice of all lines froze for 66”-98”. While there are differences among some control groups in the mean levels of freezing in CS-t sessions (e.g., female controls from the Dp16, Dp17 and Dp10 litters froze for 66, 86 and 70 s, respectively), none of these reached significance (*p* = 0.3–0.8 in pairwaise comparisons; for details, see Supplementary Tables S1–S3).

**Figure 1:**
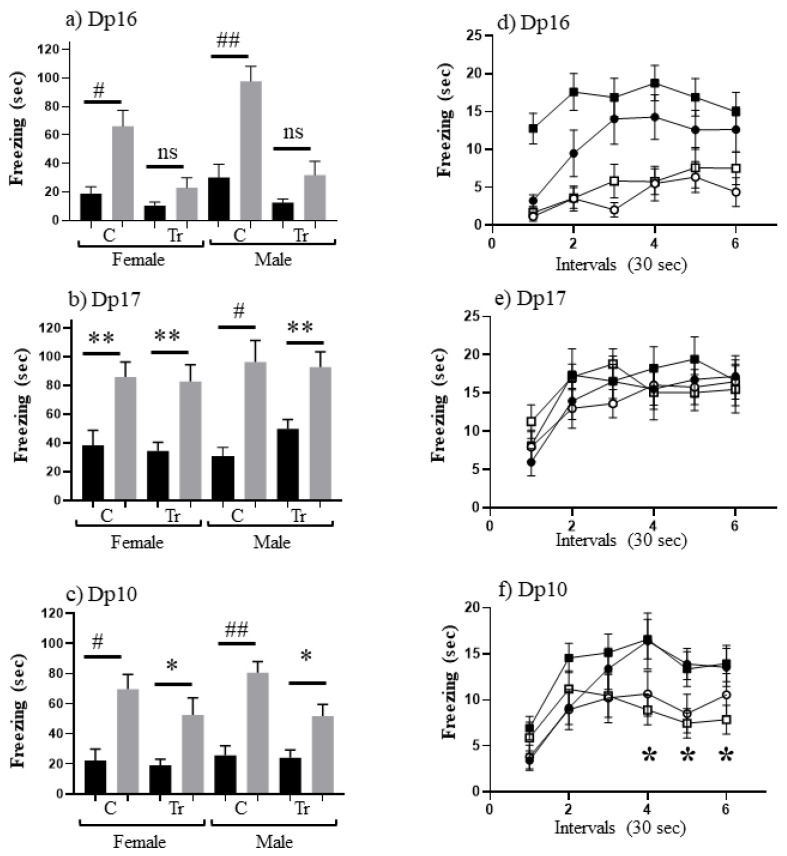
CFC performance of 3 month old mice on the C57BL/6J background. (**a**–**c**) Freezing (seconds) during 3 min of shock-context test (SC-t, black bars) and context-shock test (CS-t, grey bars). C, control; Tr, trisomic. F, female; M, male. Difference between CS-t and SC-t within sex and DS line: * *p* < 0.05–0.01; ** *p* < 0.01–0.001; # *p* < 0.001–0.0001; ## *p* < 0.0001; ns, not significant. Error bar represent the SEM. Number of animals in SC and CS, respectively: (**a**) FC, 12,11; FTr, 9,10; MC, 13,16; MTr, 10,10; (**b**) FC, 12,13; FTr, 13,16; MC, 11,12; MTr, 10,10; (**c**) FC, 11,13; FTr, 12,16; MC, 12,20; MTr, 10,19. (**d**–**f**) Freezing during CS-t 30 second intervals for the same sex and DS lines as in (**a**–**c**). Filled circles, female controls; open circles, female trisomics; filled squares, male controls; open squares, male trisomics.

To measure associative learning, we compared levels of freezing in SC-t with CS-t. Results are shown in Figure 1. Male Dp16 have been shown previously to be impaired in CFC [34], and this is demonstrated here (Figure 1a) and extended to female Dp16: i.e., as observed for trisomic male Dp16, trisomic female Dp16 do not freeze significantly more in CS-t than in SC-t. Conversely, male trisomic Dp17 have been shown to learn successfully in CFC [34]; Figure 1b shows this is true here for females as well as males. Indeed levels of freezing are not significantly different between trisomics and controls for either sex. A similar analysis for Dp10 (Figure 1c) shows that they also learn successfully because levels of freezing in CS-t are significantly greater than in SC-t for females and males. However, it is notable that trisomic Dp10 freeze for only ~50 s, levels that are ~30% lower than all control groups and trisomic Dp17 mice.

To investigate this further, we examined the time dependence of freezing in the CS-t sessions for all three lines (Figure 1d–f). Freezing levels during the three minutes were binned into 30 second intervals and corresponding interval levels were compared. For trisomic Dp16 mice, both females and males freeze significantly less than sex-matched controls (Figure 1d) for all intervals except interval 1. In contrast, trisomic Dp17 female and male mice freeze at similar levels to their respective controls during all intervals (Figure 1e). These results are consistent with data in Figure 1a,b. For trisomic Dp10 mice, females and males freeze less than respective controls during intervals 3–6. These differences are significant for male Dp10 during intervals 4–6 (*p* = 0.007, 0.02 and 0.02, respectively). This suggests that male Dp10 at this young age do not learn as robustly control mice, in this paradigm of associative learning.

### 3.2. Effects of Genomic Background on CFC Performance (Experiment 2)

The Dp16, Dp17 and Dp10 mice used here were maintained on the inbred C57BL/6J background. C57 mice are generally considered to learn well in tasks requiring a functional hippocampus, such as CFC [42]. In contrast, the DBA inbred strain has documented physical abnormalities in the hippocampus and shows impaired performance in CFC [42]. We hypothesized that a genomic background with less robust learning might uncover the effects of trisomy not evident in the stronger C57 background. Accordingly, Dp17 and Dp10 trisomic males were crossed with DBA females; F1 offspring are genetically identical (with the exception of the trisomic segments) with 50:50 C57 and DBA alleles. Performance in CFC was assessed for female and male F1 offspring, wild type and trisomic mice, at 3 months of age. We also examined female and male mice from the parent inbred DBA strain to compare their performance with C57 controls. Freezing levels for all genotypes and sexes are shown in Table 3.

**Figure 2:**
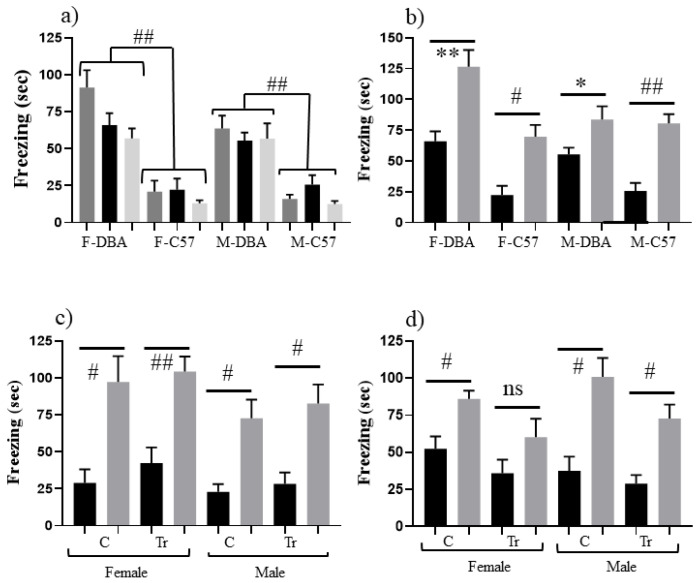
Effect of DBA/2J genomic background on CFC performance of 3 month old mice. (**a**) Freezing (seconds) during 3 min of SC training (SC, black bars), SC testing (SC-t, light grey bars) and CS training (CS, dark grey bars) of female (F) and male (M) control mice on the DBA/2J (DBA) and C57bL6/J (C57) genomic backgrounds. Freezing of DBA mice in all three measures is significantly higher than freezing in C57. (**b**) Freezing (seconds) during 3 min of shock-context test (SC-t, black bars) and context-shock test (CS-t, grey bars) for control mice on the DBA and C57 genomic backgrounds. Difference between CS-t and SC-t within sex and background. (**c**) Freezing (seconds) during 3 min of shock-context test (SC-t, black bars) and context-shock test (CS-t, grey bars) of F1 mice from crossing trisomic male Dp17 on the C57 background with female controls on the DBA background. C, control; Tr, trisomic. Difference between CS-t and SC-t within sex and DS line. (**d**) Freezing (seconds) during 3 min of shock-context test and context-shock test (CS-t, grey bars) of F1 mice from crossing trisomic male Dp10 on the C57 background with female controls on the DBA background. Difference between CS-t and SC-t within sex and DS line. * *p* < 0.05–0.01; ** *p* < 0.01–0.001; # *p* < 0.001–0.0001; ## *p* < 0.0001; ns, not significant. Error bars represent the SEM. Number of animals in SC and CS, respectively (from Table 1): (**b**) F-DBA, 8,9; F-C57, 11,13; M-DBA, 11,11; M-C57, 11,12; (**c**) FC, 7,7; FTr, 10,10; MC, 6.9; MTr, 7,9; (**d**) FC, 15,16; FTr, 9,9; MC, 9,9; MTr, 9,8.

Figure 2a shows that both female and male DBA control mice freeze significantly longer in SC, SC-t and CS than C57 control mice: >50 s vs. ≤25 s, respectively (*p* ≤ 0.001–*p* = 0.002), suggesting that DBA mice are more sensitive to both novelty and shock. Figure 2b shows that DBA control mice nevertheless still learn successfully because there is a significant difference between time spent freezing in CS-t and SC-t. This result differs from prior reports of DBA failure in CFC [42]. This difference is likely due to the assessment here of learning only (at 60 min post training), while previous experiments assessed learning plus memory at 24 h. This suggests that DBA mice can indeed learn the context but likely fail to consolidate the memory. For assessment of the F1 hybrids, Figure 2c shows that both female and male Dp17 F1s learn successfully, but while male trisomic and control Dp10 F1s learn successfully, female trisomic Dp10 F1s do not learn (Figure 2d).

### 3.3. Performance in CFC at ~18 Months of Age (Experiment 3)

**Figure 3:**
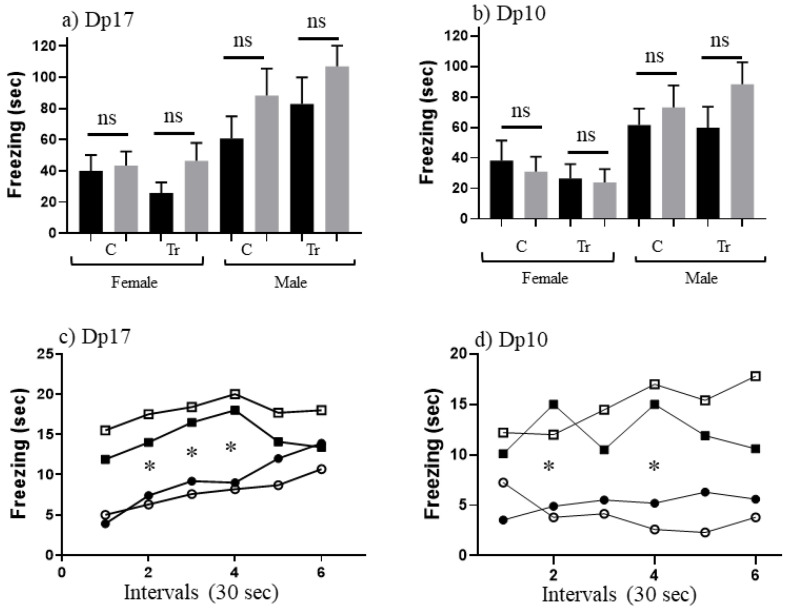
CFC performance of 18 month old mice. (**a**,**b**) Freezing (seconds) during 3 min of shock-context test (SC-t, black bars) and context-shock test (CS-t, grey bars). C, control; Tr, trisomic. F, female; M, male. Difference between CS-t and SC-t within sex and DS line: ns, not significant. Error bars represent the SEM. Number of animals, SC and CS, respectively (from Table 1): (**a**) FC, 7,8; FTr, 7.6; MC, 9,6; MTr, 10,11; (**b**) FC, 10,11; FTr, 10,10; MC, 8,10; MTr, 9,13, (**c**,**d**) Freezing during CS-t 30 second intervals for the same sex and DS lines as in (**a**,**b**). Filled circles, female control; open circles, female trisomic; filled squares, male controls; open squares, male trisomics. *, significant difference between females and males of corresponding genotypes, *p* < 0.008–0.04.

Table 4 shows freezing levels in SC and CS training and test sessions for naïve Dp17 and Dp10 aged to ~16–24 months. For female mice, both Dp17 and Dp10, controls and trisomics, levels of freezing in SC-t are 26”–55”, which is generally higher than the 19”–39” seen in the corresponding 3 month old mice. Similarly levels for male Dp17 and Dp10, control and trisomic mice, range from 60”–83”, with each higher than levels for corresponding 3 month old mice. This is not a general age-related decrease in locomotion or exploratory behavior, because mice in a “no shock” (NS) group still froze only briefly, for ~6–13 s, and those in the CS training group froze for only 7”–11”, not different from CS levels in 3 month old mice. Together, these data suggest that both controls and trisomic mice at this age are more sensitive to the electric shock experienced immediately upon being placed in the novel cage than are 3 month old mice. In the CS-t groups, Dp17 and Dp10, controls and trisomics, levels of freezing are 35”–56”, which is lower than freezing levels in successful learning in the younger mice who all froze for >70 s (with the exception of trisomic Dp10). This, coupled with the increased freezing in SC-t, results in no significant difference between SC-t and CS-t (Figure 3a,b), i.e., there is no evidence of associative learning in this paradigm in these older mice.

We next examined the time dependence of freezing in CS-t (Figure 3c,d). Female mice, both control and trisomic, show lower levels of freezing than male mice of the corresponding genotypes. This is significant for female controls at intervals 4–6 (*p* = 0.018, 0.007 and 0.003, respectively) and Dp10 trisomic females at intervals 2–6 (*p* = 0.005, 0.066, 0.002, 0.005 and 0.065, respectively).

## 4. Discussion

Mice are a practical model animal for the study of many human genetic diseases. DS is particularly complicated genetically because it involves an extra copy of an entire chromosomal arm that encodes several hundred genes [21]. Modeling DS in mice is further complicated by the need to study the triplication of segments of the three different mouse chromosomes that contain Hsa21 orthologous genes [43]. To date, the orthologous segment of Mmu16 has received almost exclusive attention in DS research. This is not because the encoded genes have been demonstrated to be more relevant to the DS phenotypes than orthologs on Mmu17 and Mmu10, but only because the first viable trisomic mouse model, the Ts65Dn, was trisomic for a large part of the Mmu16 segment, displayed many DS relevant features, and was widely available more than 15 years before trisomic models for the Mmu17 and Mmu10 regions were constructed [23,29,34]. Thus, it is important to focus some effort on mouse models of these latter regions. Other common limitations of mouse studies of DS include examination of a single age and single genetic background, and the almost exclusive use of male mice. The latter has occurred, not only because it was an accepted standard in the field, but also because challenges in breeding the Ts65Dn (males have very low fertility, females have few and small litters with <~30% trisomic pups [33]) result in females generally being reserved as breeders.

Here, we examined learning in three mouse models of DS, the Dp16, Dp17 and Dp10, which together are trisomic for all Hsa21 orthologs mapping to mouse chromosomes 16, 17 and 10, respectively [34]. We used a CFC training protocol, a single shock of 0.7 mA, which is less intense than in other DS mouse studies, where either a stronger shock, of 1.0 mA, or multiple shocks, have been used [34,36,39]. In addition, mice were tested for learning 60 min after training, instead of the more common 24 h later. The time of 60 min was selected to facilitate future protein expression analyses; it has been shown that the MAPK pathway must be activated during this time for learning to occur [41,44,45]. This also means that learning is assessed and not memory.

In all experiments, we used separate cohorts of female and male mice. The existence of sex differences, in people, in the incidences of specific neurological disorders and in drug responses has become increasingly clear [46,47,48,49,50]. Sex differences in rodents have been well documented in learning strengths and weaknesses, in learning strategies, and in the molecular responses and features subserving learning and memory [51,52,53]. We also examined the effects of genetic background. Most commonly mouse model studies use inbred lines, i.e., genetically identical individuals. This homogeneity can mask differences that may be seen when studies are extended to genetically highly diverse human populations. The DBA/2J strain was chosen here for investigation of learning in F1 hybrids because it has structural abnormalities in the hippocampus [54,55] and shows deficits in hippocampal tasks, including CFC [42]. Here we found no differences in CFC performance between females and males in the Dp16 and in Dp17 models on the original C57BL/6J background, i.e., similar to male trisomic Dp16, female trisomic Dp16 failed, and similar to male trisomic Dp17, female trisomic Dp17 learned as well as controls. Performance of the Dp17, both females and males, was also unaffected by presence of the 50% DBA background (Dp16 were not tested). We note that results here for male Dp16, i.e a general failure to recognize the context, are consistent wth those seen in CFC testing of the popular Ts65Dn mice that are trisomic for a reduced segment of the Hsa21 orthologous region of Mmu16 [25,26]. Female Ts65Dn have not been tested in CFC.

In contrast to the Dp16 and Dp17, Dp10 mice showed sex-specific deficits, with contributions from the genetic background. On the C57 background, while females and males both learned successfully, male Dp10 showed a significantly lower level of freezing than male controls. This indicates a subtle impairment, unobserved in prior studies and uncovered here by the use of a less intense training protocol. While its practical importance remains to be determined, it would be consistent with an observation that children with DS may require more intensive learning strategies, longer learning times or more repetitions in order to learn as effectively as typical children in some tasks. It is noteworthy that male children with DS have been reported to show greater levels of cognitive impairment than females in some tasks [56,57,58].

The DBA genetic background differentially influenced trisomic females and males. Female Dp10 F1s were impaired in CFC, while male Dp10 F1s, in contrast to the C57 background, learned successfully, freezing at levels similar to controls. Thus, the hippocampal abnormalities of the DBA strain coupled with trisomy of the Mmu10 region uncovered a female deficit while rescuing males from the impairment seen on the C57 background.

The Mmu10 region encodes orthologs of 39 Hsa21 classical protein coding genes [21]. Several among these, when mutated or altered in expression level, have been shown to influence brain development and function, including learning and memory (reviewed in [21,59]. In addition, four of these genes are the only Hsa21 genes known to have sex-specific functional features. One of these is PRMT2, a protein Arg methyl-transferase that modifies and directly activates estrogen and progesterone receptors and the thyroid hormone receptor B, and indirectly activates the androgen receptor [60,61]. Another is SUMO3, a member of the small ubiquitin-like modifier protein family that modifies the Nuclear Receptor Co-repressor, NCOR2, which in turn inhibits activities of estrogen, progesterone and androgen receptors [62,63,64]. Thus, overexpression of these genes in trisomy could have consequences for sex hormone signaling and regulation.

Two other Mmu10 Hsa21 orthologs show sex-specific phenotypes as null mutations. The adenosine deaminase, ADAR2, regulates activities of a serotonin receptor and N-methyl-D-aspartate (NMDA) and gamma-aminobutyric acid (GABA) receptors through pre-mRNA editing (reviewed in [65]). An ADAR2 knockout impairs acoustic startle response in males but not females [66]. TRPM2 (Transient Receptor Potential cation channel subfamily M member 2) is a Ca-permeable cation channel activated by oxidative stress. Inhibition protects from ischemia through modulation of NMDA receptor subunit expression; knockout protects males but not females from effects of ischemia through a mechanism that involves the androgen receptor [67,68]. While observations from knockouts cannot predict the effects of over expression expected in trisomy, the sex-specific phenotypes suggest that trisomy might also differently affect females and males.

Sex and trisomy-specific differences in protein expression have been demonstrated in the hippocampus, cortex and cerebellum of 8-month-old naïve Dp10 and littermate controls [59,69]. Proteins measured include multiple components of the MAPK, MTOR and apoptosis pathways, immediate early gene proteins, and several subunits of ionotropic glutamate receptors, in addition to other proteins known to function in synaptic plasticity and intellectual disability. Of the ~100 proteins measured, approximately half were expressed at levels significantly higher in the hippocampus of female control mice compared with male controls. Superimposed on these normal sex differences, trisomy of the Mmu10 region differentially perturbed expression in females and males; Dp10 females showed the most differences from female controls in cerebellum and males showed the most perturbations in the hippocampus. Which of these baseline abnormalities are deleterious, and which are potentially compensatory, is not known; how they would respond to the stimulation to learn remains to be determined.

Although protein measurements in [59,69] were made in naïve mice and at 8 months of age vs. the 3 and 18 months examined here, expression abnormalities of Hsa21 orthologs is interesting. Proteins encoded by three genes trisomic in the Dp10 were measured, two with natural sexually dimorphic features and one without. Levels of PRMT2 were unaffected by sex or trisomy in hippocampus but in cerebellum were affected by both: in control mice, females had significantly lower levels than males, but in trisomic mice, females had levels twice that of males. Levels of ADAR2 were sexually dimorphic only in hippocampus and only in trisomic mice: in male Dp10 mice, ADAR2 levels were elevated by 50% compared with control males, consistent with an extra copy of the gene, however in female Dp10, levels were not different from controls. In contrast to PRMT2 and ADAR2, levels of the S100B protein, a calcium binding protein with roles in inflammation, showed no sex differences in controls or Dp10 and was uniformly elevated in cerebellum, hippocampus and cortex in trisomy, consistent with gene dosage. Thus, proteins with known sex differences in function may be associated with more complex perturbations in trisomy showing novel sex differences in expression with brain-region specificities. Additional experiments are needed to determine the contributions of perturbed expression of sexually dimorphic Hsa21 orthologs to the subtle learning deficits in both female and male Dp10 mice.

## 5. Conclusions

The Dp10 mouse model of DS, at 3 months of age, is impaired in learning in CFC. This impairment was uncovered, using a mild training protocol, in male Dp10 on the C57 genetic background and in female Dp10 on the C57/DBA F1 hybrid background. In contrast, there were no sex differences in CFC performance using this protocol in the Dp16 mice on the C57 background or in the Dp17 mice on either the C57 or the C57/DBA backgrounds. Thus, to identify genes contributing to learning and memory deficits in DS, it is advisable to investigate female as well as male mice on genetic backgrounds with different cognitive strengths and weaknesses. CFC is not an appropriate associative learning task for mice of either sex ~18 months. Expanding preclinical evaluations of drug treatments to mice of both sexes, multiple ages and genetic backgrounds is expensive in time and resources but could contribute to more effective outcomes of clinical trials.

## Figures and Tables

**Table 1:** Numbers of mice analyzed in each cohort of each experiment.

	Female Control			Female Trisomic			Male Control			Male Trisomic		
	SC	CS	NS	SC	CS	NS	SC	CS	NS	SC	CS	NS
Experiment 1												
Dp16	12	11		9	10		13	16		10	10	
Dp17	12	13		13	16		11	12		10	10	
Dp10	11	13		12	16		12	20		10	19	
Experiment 2												
DBA	8	9					11	11				
DBA X Dp17	7	7		10	10		6	9		7	9	
DBA X Dp10	15	16		9	9		9	9		9	8	
Experiment 3												
Dp17	7	8		7	6		9	6	5	10	11	5
Dp10	10	11	5	10	10	5	8	10	7	9	13	7

**Table 2:** Average time freezing in CFC groups of three-month-old mice. Mice were placed in a novel cage for three minutes in a training session (SC or CS) and a testing session (SC-t or CS-t); training and testing were separated by 60 min in the home cage. SC, shock-context; mice received the electric shock during the training session immediately on being placed in the novel cage. CS, context-shock; mice received the electric shock during the training session after three minutes in the novel cage. Times are provided in seconds. For numbers of mice in each group and SEM of times, see Figure 1.

			SC	SC-t	CS	CS-t
Dp16	Female	C	13	18	13	66
		Tr	8	10	10	23
	Male	C	14	30	7	98
		Tr	7	12	7	32
Dp17	Female	C	29	39	15	86
		Tr	35	35	11	83
	Male	C	18	31	11	96
		Tr	19	50	31	93
Dp10	Female	C	19	22	13	70
		Tr	17	19	11	53
	Male	C	16	26	13	81
		Tr	17	24	14	52

**Table 3:** Average time freezing in CFC in DBA/2J inbred mice and F1 hyrbids. Mice were placed in a novel cage for three minutes in a training session (SC or CS) and a testing session (SC-t or CS-t); training and testing were separated by 60 min in the home cage. SC, shock context; mice received the electric shock during the training session immediately on being placed in the novel cage. CS, context-shock; mice received the electric shock during the training session after three minutes in the novel cage. Times are provided in seconds. For numbers of mice in each group and SEM of times, see Figure 2.

			SC	SC-t	CS	CS-t
DBA/2J	Female	C	91	66	60	127
	Male	C	64	55	57	84
DBA X Dp17	Female	C	15	29	14	97
		Tr	21	22	11	104
	Male	C	16	23	11	73
		Tr	21	28	14	83
DBA X Dp10	Female	C	37	52	13	86
		Tr	23	37	13	60
	Male	C	35	38	15	101
		Tr	26	29	17	73

**Table 4:** Average time freezing in CFC in ~18-month-old mice. Mice were placed in a novel cage for three minutes in a training session (SC or CS) and a testing session (SC-t or CS-t); training and testing were separated by 60 min in the home cage. SC, shock-context; mice received the electric shock during the training session immediately on being placed in the novel cage. CS, context-shock; mice received the electric shock during the training session after three minutes in the novel cage. Times are provided in seconds. NA, not available. For numbers of mice in each group and SEM of times, see Figure 3.

			SC	SC-t	CS	CS-t	NS	NS-t
Dp17	Female	C	25	55	10	56	NA	NA
		Tr	15	26	11	46	NA	NA
	Male	C	28	61	7	88	11	39
		Tr	53	83	11	107	9	12
Dp10	Female	C	24	38	13	31	9	21
		Tr	20	38	8	24	6	17
	Male	C	44	62	11	73	13	25
		Tr	19	60	9	88	8	23

## Data Availability

Data are provided in Supplementary Tables S1–S3 available online and from the authors.

## Supplementary Materials

The following are available online at https://www.mdpi.com/article/10.3390/genes12101528/s1, Table S1. CFC data for all 3 month old groups; Table S2. CFC data for 18 month old groups; Table S3. Mouse information.
